# Supplementary material for: UBC Gene Family Analysis in Salvia castanea and Roles of ScUBC2/5 Genes under Abiotic Stress
Source: Plants (Basel). 2024 May 14;13(10):1353. doi: 10.3390/plants13101353 (PMC11125094; doi:10.3390/plants13101353)
Supplement: Supplementary file 1 [file plants-13-01353-s001.zip › plants-2950450-supplementary.pdf]

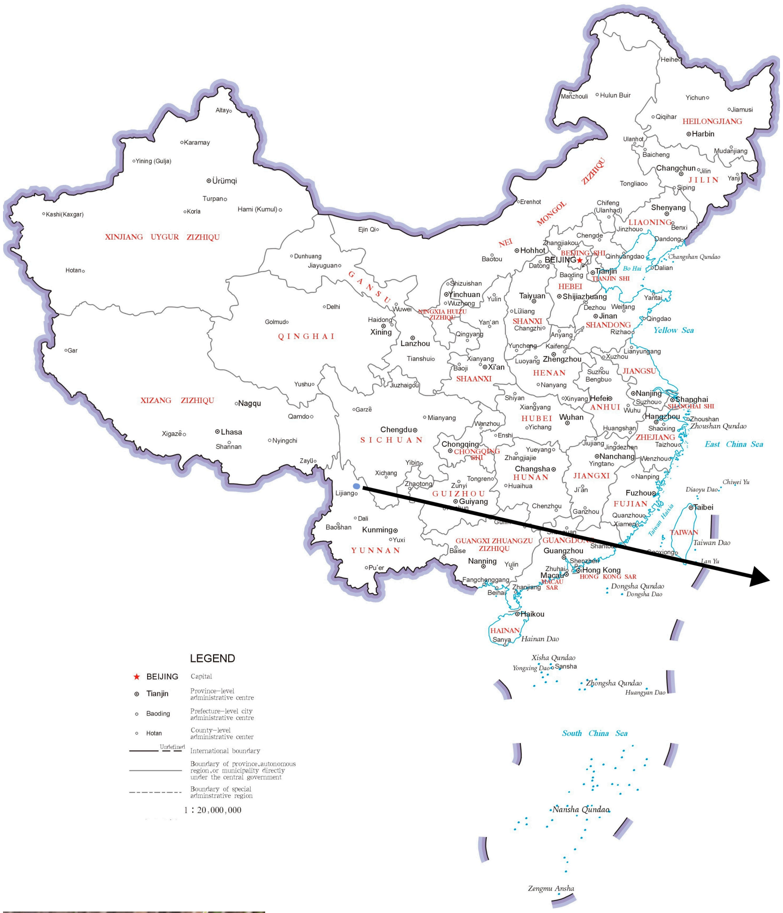

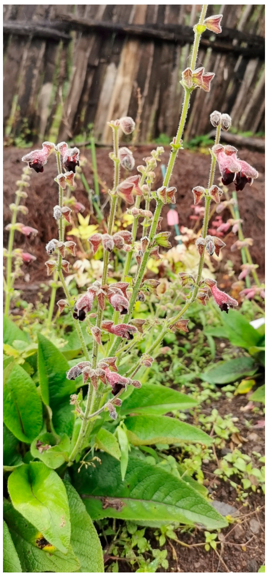

Figure S1. Jade Dragon Snow (Yulong) Mountain in China and the morphological characteristics of *Salvia castanea* in the habitat. Longitude, latitude, and altitude: 100°15′ E, 27°9′ N, and 3052 m.
